# Unusual Condition of the Skin and Kidneys Following an Operation for Appendicitis1Read at the seventy-ninth annual meeting of the Medical Society of the County of Erie, January 9, 1900.

**Published:** 1900-05

**Authors:** W. G. Taylor

**Affiliations:** Buffalo, N. Y.


### UNUSUAL CONDITION OF THE SKIN AND KIDNEYS FOLLOWING AN OPERATION FOR APPENDICITIS.[1]

By W. G. TAYLOR, M. D., Buffalo, N. Y.

WE ARE always interested in usual conditions because of their practical importance, but unusual conditions have an additional scientific interest. If all my patients who suffer from appendicitis had before them an array of symptoms like the following case, I would with caution advise an operation, unless the life of the patient was despaired of.

On January 7, 1896, I was called to see Miss B., aged 18—previous health good—who was suffering from appendicitis with the usual symptoms of the first stage. Dr. Frederick was called in consultation and an operation was advised and performed within eight hours after seeing the patient. The appendix was found enlarged and injected with a considerable number of adhesions already formed. Patient made apparently an excellent recovery, but after being up, however, for a month she began to have pain in the incision, at first at irregular intervals, but later all the time; it was a sharp pain extending through the back and down the right limb. Patient could not lie on the left side because of an increase of pain. She began to lose appetite and spirits, finally the stomach became irritable and it refused everything. Every remedy know for pain as drugs, counter-irritants, thermo-cautery, etc., were applied with no effect. Eight different physicians saw the case, but no definite diagnosis could be made, but opinions were expressed that it might be a nerve sewed in the wound or an adhesion attached to the under surface of the scar.

On the 5th of May, four months after the original operation, a dissection was made of the scar and a nerve removed which was thought to be the cause of the pain, but much to the disappointment of all concerned the pain continued as severe as ever. The patient at this time was able to keep only liquid beef peptonoids on the stomach, was despondent and life was a burden. Everything was done for the relief of the patient, as after the first operation, but of no avail. I had an opportunity to watch the case very closely, and it was my firm conviction that it was an adhesion, but I did not want to advise reopening the peritoneal cavity unless it was absolutely necessary, for it meant more danger and several weeks in bed to an already bed-ridden patient, and, as you all know, such advice was not the most encouraging thing in the world in mid-summer and to one who had given up all idea of getting relief.

After a great deal of urging, and a promise of relief, the patient consented to a third operation, which was done on July 7th, six months after the first operation. An adhesion was found attached to the under surface of the scar involving the omentum, about the size

1. Read at the seventy-ninth annual meeting of the Medical Society of the County of Erie, January 9, 1900.

of a piece of twine. This was removed and she has had no pain from that day to this.

Patient did well for six weeks, or as well as could be expected under the circumstances, when her stomach began to give her trouble; patient was nauseated more or less all the time and vomited several times daily. Suffered from anorexia, constipation and amenorrhea. Has menstruated but once since her last operation and then one month later. I never saw a patient who lived on so little food, the slightest thing seemed to produce fermentation. She suffered in this way for two or three months, but improved somewhat under stomach sedatives and antiseptics, but more particularly lavage of the stomach. Patient continued, however, in miserable health during the winter of '97, and had more or less nutritional disturbance.

In the spring of 1897 the kidneys, skin and bowels showed marked evidences of disturbed function. I asked for a twenty-four hour sample of urine and much to my surprise found the specific gravity 1060, the quantity for twenty-four hours being one-half pint. On the 16th of June she only passed five drams during twenty-four hours. June 18th, eight ounces; specific gravity, 1080. At this time there appeared all over her body an eruption, which to my mind showed that the skin was trying to do the work that the kidneys were expected to do. Dr. Grover Wende was called in to see the case and pronounced the condition hydrocystoma. (His report I will submit later.) This condition of high specific gravity continued for two months. July 19th, specific gravity was 1100; July 22d, specific gravity was 1090. The examination of the 1100 sample was as follows: Quantity for twenty-four hours, twenty-four ounces. Consistency, mucilaginous, poured like syrup; reaction, alkaline; color, amber; no albumin or sugar; total urea, 300 grains. Microscope showed amorphous and triple phosphates in abundance, but nothing to account for the high specific gravity. On July 23d quantity began to increase—one and one-half pints in twenty-four hours; specific gravity, 1070; July 24th, one pint; specific gravity, 1090; July 25th, one and one-half pint; specific gravity, 1070. At this time the eruption began to improve, but high specific gravity continued until about August 15th, at which time samples were delivered to Dr. J. A. Miller, for more thorough examination. His report is as follows:

Referring to my records with regard to the urine of Miss B., I find that the first sample was delivered to me on the 14th of August, 1897. The analysis was, viz.: Quantity per twenty-four hours, unknown; specific gravity on the filtered sample=1081.8. The gravity was taken with the Westphal balance. Reaction, alkaline, due to ammonia. Color, amber; albumin, sugar, bile and bile acid, absent; indican, slightly above normal. Urea,=1.25 grams per 100 c.c. Chlorine and sulphates, largely above normal. Earthy phosphates, in traces only. Alkaline phosphates above normal. The microscopical examination revealed the presence of triple phosphate crystals in large quantity, also the presence of crystals of calcium phosphate, ammonium urate, together with mucus and bladder, vaginal and urethral epithelium.

Not feeling satisfied with the above, I called for a full twenty-four hour sample. The analysis is viz.: Quantity, 465 c.c.; color, amber; odor, ammoniacal; reaction, alkaline; specific gravity by Westphal=1094.4. The total solids calculated from the gravity would be 102.27 grams in 465 c.c., or twenty-four hours. This quantity is no doubt too large, as the determination of total solids by direct evaporation and weighing shows only 72.16 grams for 465 c.c., or twenty-four hours. Urea,=5.12 grams per twenty-four hours. Uric acid,=0.14 grams per twenty-four hours. Chlorine,=2.43 grams and sulphuric acid ($SO_3$) 0.46 grams, phosphoric acid,=0.35 grams per twenty-four hours. A microscopical examination on this sample was not made. After a careful examination to ascertain the cause of the high gravity, I concluded that it was due to the presence of glycogen.

The method pursued for the isolation of glycogen from the urine was as follows: The urine was evaporated to a syrupy consistency and filtered; a solution of caustic potash was then added, followed by absolute alcohol, until no further precipitation occurred. The precipitate was carefully washed with alcohol, and then dissolved in acetic acid, filtered and again precipitated with absolute alcohol and thoroughly washed with alcohol. After drying over sulphuric acid the glycogen was dissolved in water. The solution thus obtained was strongly opalescent; it gave with iodine solution a red color, with basic lead acetate a white precipitate. Treated with dilute hydrochloric acid it reduced Fehling's solution after neutralization of the acid.

On November 4, 1897, at my request, patient brought a good-sized sample. The sample was faintly acid in reaction; color, amber; in appearance clear except for the presence of a slight mucus cloud. The quantity passed was 80 c.c., and had a gravity of 1023. It contained 1.6 grams of urea.

On the 22d day of April, 1898, I received another twenty-four hour sample. The total quantity was 600 c.c.; color, amber; turbid in appearance; odor, urinous; specific gravity, 1019; reaction, neutral; albumin, sugar, bile and bile acid, absent; urea, =9 grams in twenty-four hours; uric acid, =0.16 grams; indican, normal; earthy and alkaline phosphates, slightly above normal; sulphates, normal; chlorine markedly in excess. The microscopical examination showed the presence of organic débris, mucus corpuscles, bacteria in abundance, a few red blood corpuscles, a small amount of vaginal, bladder and urethral epithelium; uric acid crystals in fair abundance, ammonium urate, a few triple phosphate crystals and fungus.

I did not find any glycogen in this sample, which owing to the normal gravity was to be expected. The presence of glycogen in the urine is rare, there being very few references in the literature thereto. Glycogen itself is a white amorphous powder, tasteless and odorless. It dissolves in water to form an opalescent solution. It is insoluble in alcohol and in ether. Does not reduce Fehling's solution.

The treatment given with an aim to bring the kidneys back to a normal condition was steam baths, diuretin, infusion digitalis and malt as a tonic. The bowels came to the rescue in September and she had a most violent dysentery for a week. For the first two days bowels passed off almost hourly with large quantities of blood, but at the end of the week had only two or three movements daily. Improvement set in from this time very rapidly, and by November 4, as Dr Miller states in his report, she had a normal urine. This is the highest specific gravity on record as far as I know. I wrote Prof. Osler, of John Hopkins' University, at the time, and he replied that the highest specific gravity that he ever saw was 1060, and in this case the patient was surreptitiously putting sugar in her own urine. If it was sugar that was producing the highest specific gravity in this case we might be sceptical, but very few of us, if any, have seen glycogen, much less to know anything about it, besides she was a very sick young woman, and her symptoms were in keeping with imperfect action on the part of the liver, skin, kidneys, stomach and bowels.

Dr. Grover Wende's diagnosis of the case was:

"The condition of the face, arms, chest and abdomen involve many clinical points in common with hydrocystoma. The number of lesions was not to be estimated—these were distributed with wonderful symmetry. The lesions upon the face varied in size from a pin's head to a split pea; the entire surface was covered with the small cysts, but they were most abundant below the level of the orbits, over the bridge of the nose and close to the nares. At the last named point the lesions were conjoined and appeared to be confluent, although a close inspection showed that this was not the case. Nowhere upon the face was to be discovered any inflammation of the skin or any alteration of the color of the surface surrounding the lesions, which appeared to be deeply imbedded in the skin. The vesicles were firm to the touch. The distribution over the chest was quite general and extended over the upper part of the abdomen. Most of the lesions were like those on the face, varying in size from a pin's head to a lentil; scattered here and there among the nonhyperemic lesions were some twenty-five distinct vesicles about the size of a bean which displayed marked redness at the base and appeared inflammatory. There were also a number of cysts distributed upon both arms near the shoulder, but no inflammatory lesions were to be discovered among them.

The patient states that during the time when her kidneys were secreting so small an amount of urine she was subject to profuse sweating after undue exercise. The acute attack already described began upon the nose and immediately following affected the other regions. This condition extended over a period of about four months, during which time exacerbations twice resulted. The lesions were slow in disappearing—some of them lasted for weeks. An increased influence was experienced upon exposure to heat or after partaking of hot fluids. Unfortunately in the case under consideration there was no microscopical examination made of the tissue or even the reaction of the cyst contents was not taken. I believe the condition to be nearly identical with the disease first described by Dr. A. R. Robinson as a special form of sudamina under the name of hydrocystoma. (*Journal of Cutaneous and Genito-Urinary Diseases*, Vol. II., p. 295.) The eruption of this case differs clinically from that brought to notice by Robinson, in the fact that while his was limited to the face, this appeared on other portions of the body. Besides, the many inflammatory lesions to be seen upon the chest constitutes a feature foreign to the ordinary manifestations of hydrocystoma. The present case cannot be regarded as a typical one from an etiological standpoint, by reason of the deficient action of the kidneys, betraying a condition clearly abnormal.

In making the diagnosis of hydrocystoma in this case it was based upon the characteristic appearance of the lesions as they appeared upon the face and the want of tendency to spontaneous rupture, also the long duration of them. Diseases by which you would be likely to be confused are so few and so radically different in their appearance that there is little excuse for false conclusions. The difference between sudamina crystallina and the condition here described need hardly be considered. The lesions in sudamina are so superficial and so readily wiped off that they could not possibly be confounded with the immovable cyst of hydrocystoma and with pompholyx, which is an acute inflammatory infection of the head and feet, followed by a dry red surface, resulting from a desquamation of the epidermis. The greater resemblance between hydrocystoma and the adenoma of the sweat glands when confined to the face, lies in the fact that in the latter disease the lesions are solid, grow slowly and do not vary in size. The difference between the same condition and eczema, is that the latter tends to rupture and to leave a moist, exuding surface, while the vesicles are pin-head size, usually of uniform dimensions and always accompanied by redness of the skin and the subjective sensation of itching.

As regards this case of hydrocystoma, the question naturally arises, what is the cause of the affection? In Dr. Robinson's well-known work these cysts are alleged to be due to a disordered function of the sweat-glands. In the majority of his cases there was always a condition of hyperidrosis or some sort of derangement of the excretory glands, limited to the face and appearing in persons who were exposed to extreme heat. Laundresses are particularly liable to this condition. Hallopeau was the first to take into consideration other causes, by calling attention to the fact that the affection was more marked during the menstrual period and seemed to be equally influenced by high temperature and violent emotions. Many other cases have since been recorded in which the nerve influence was supposed to have something to do with the origin of the disease. It is quite easy to understand that the hyperidrosis acted as the exciting cause in the present case, as the kidneys were eliminating but a small amount of fluid, requiring the skin to perform nearly all their legitimate work. From the history, as here given, and the observations made by Dr. Taylor, there is no doubt that the patient under consideration suffered from severe functional nervous derangement, so often found in young women. But why the vasomotor nerves regulating the caliber of the bloodvessels which supplied the skin area, in which the eruption was like that of the face, as in nearly all the cases heretofore reported, should allow the cysts to be limited to the facial region, or in this case should be limited to the regions mentioned, is a problem yet to be solved. My theory is that some cases of hydrocystoma are due to neurosis and that the face, being rich in nerves and blood supply presents a favorable field for the operation of the disease. Yet that does not explain the present case, since the amount of work performed by the skin, and the presence of other well-known manifestations of neuroses are so clearly abnormal. Whether all this will help us much etiologically is open to doubt. What we still want to know is what is the agent which produced the diseased function?

Cause and effect in this case were very beautifully exemplified. This patient went through a terrible mental and nervous strain, and I take it that the organic nervous system was largely accountable for the state of affairs. As a result of the shock to the organic nervous system, the digestive tract suffered first and no doubt primarily the liver. We all know that it is the great eliminator of the food stuffs, and they no doubt were thrown into the circulation in an imperfect condition for absorption, and first of all the stomach undertook to eliminate, which was shown by the anorexia and irritability. As an additional means of retention of the effected products was the amenorrhea and obstinate constipation. Elimination must take place to rid the system of this poison, or a toxemic condition would develop which would sooner or later claim our patient. Fortunately, the three great means of elimination, nature's own remedy, came to the rescue—the skin, kidneys and bowels.

The patient at the present time, three years after the first operation, is still under treatment; she suffers from constipation, does not nourish well, but holds her own in weight, has not menstruated, and as a result has more or less backache. Aside from the above she is in comparative health. I submit this paper as a report of the case under consideration, and would invite a discussion as to the causes leading up to the conditions present in this patient.

199 Franklin Street.

